# Case Report: Successful Control of Pulmonary Metastatic Pheochromocytoma With Iodine-125 Seed Implantation

**DOI:** 10.3389/fendo.2021.714006

**Published:** 2021-08-09

**Authors:** Hongbing Shi, Chao Wang, Weiguang Qiang, Bai Sun, Hao Wang, Ye Yuan, Wenwei Hu

**Affiliations:** Department of Oncology, The Third Affiliated Hospital of Soochow University, Changzhou, China

**Keywords:** pheochromocytoma, metastasis, ^125^I seed implantation, brachytherapy, interventional therapy

## Abstract

Pheochromocytoma with lung metastases is rare in clinics, and the prognosis of metastatic pheochromocytoma is generally poor. In this case, a 57-year-old woman who presented with hypertension and palpitations was diagnosed with left adrenal pheochromocytoma with lung metastasis in 2010. The patient received left adrenalectomy for pheochromocytoma 10 years ago, but pulmonary lesions had significant progression 7 years ago. The patient was treated with iodine-125 (^125^I) seed implantation for pulmonary lesions. All of the 5 pulmonary lesions achieved partial response 6 months later, further shrank 1 year later, and were successfully controlled for 7 years. This case indicated that ^125^I seed implantation could be an alternative local therapy for metastatic pheochromocytoma in the lung.

## Introduction

Pheochromocytoma is a rare endocrine neoplasm that arises from chromaffin cells of the adrenal medulla. Approximately 10% of pheochromocytomas are metastatic pheochromocytomas, and only metastases are the proof for malignant pheochromocytomas (1). Metastases are commonly located in the liver, bones, lymph nodes, and lungs (2, 3). For patients with symptomatic metastases or significant disease progression, several local or systemic therapies are available to reduce tumor burden, but standard curative management has not been established so far. Cytoreductive resection, thermal ablation, and external beam radiation therapy (EBRT) are recommended for local management of metastatic pheochromocytomas. Comparing with those therapies, the use of radiopharmaceutical agents or chemotherapy is more suitable for widespread and rapidly progressive metastatic pheochromocytomas (4, 5).

Iodine-125 (^125^I) seed brachytherapy is a modality of continuous low-dose-rate irradiation, in which a dose of <1 Gy per hour is continuously delivered by radioactive sources. ^125^I seed implantation is the most common approach of ^125^I seed brachytherapy and it has been performed in various types of locally recurrent or metastatic carcinomas in recent years (6–8). The efficacy of ^125^I seed implantation in pheochromocytomas has not yet been reported. Herein, we report a case of a patient with metastatic pheochromocytoma in bilateral lungs, who was treated with ^125^I seed implantation 7 years ago and achieved good efficacy.

## Case Report

**Figure 1:**
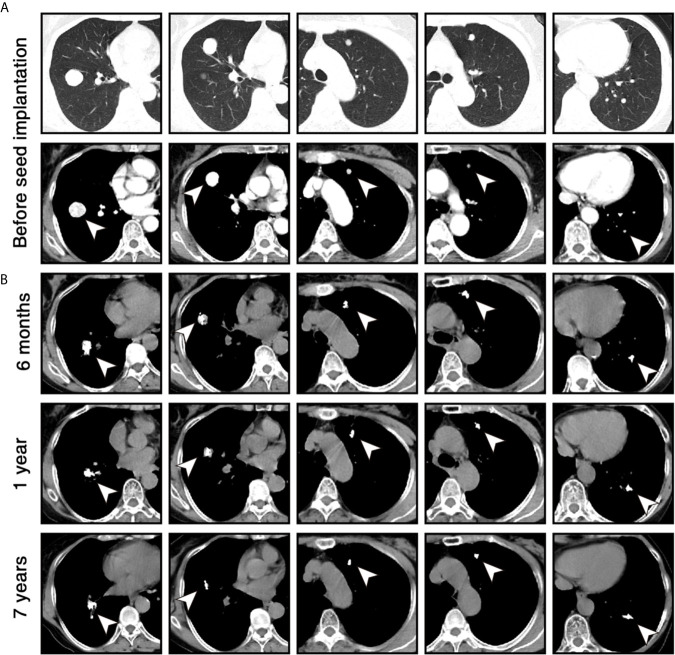
Chest computed tomography (CT) scans of the patient during the follow-up. **(A)** Before the ^125^I seed implantation, contrast-enhanced CT images in lung windows (upper) and mediastinal windows (lower) show 5 enhanced nodules (white arrowheads) in both lungs. **(B)** Six months, 1 year, and 7 years after ^125^I seed implantation, unenhanced CT images in mediastinal windows show that volume of lesions was significantly reduced within 1 year and remained stable for 7 years. High-density ^125^I seeds were located in the original sites of the lesions.

**Figure 2:**
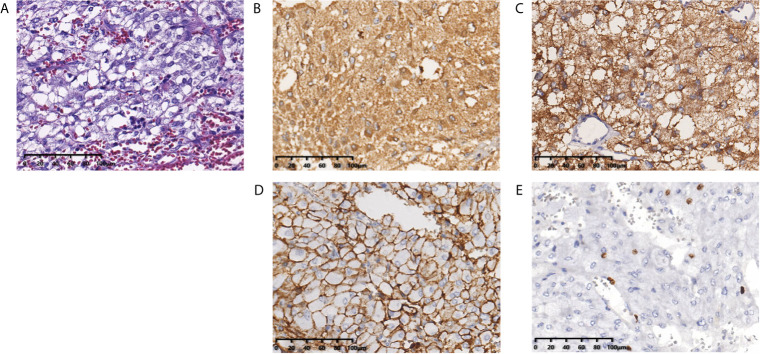
Histopathological examination shows the pulmonary nodules were metastatic pheochromocytoma. **(A)** Hematoxylin and eosin staining of biopsy specimens shows nests of tumor cells separated by vascular septa (Zellballen). **(B)** Chromogranin and **(C)** synaptophysin immunostaining is diffuse strong positive in the tumor cells. **(D)** CD56 immunostaining is positive in the membrane of the tumor cells. **(E)** Ki-67 immunostaining shows approximately 5% of cells are positive. Original magnification × 200.

In September 2010, a 57-year old woman with hypertension and palpitations was found with a mass in her left adrenal medulla by ultrasound on the regular checkup. She was referred to our hospital 1 month later. Computed tomography (CT) scans displayed a heterogeneous solid tumor, 6.9 × 5.5 cm in size, in the left adrenal medulla, as well as two nodules at less than 1 cm in the right lung (one in the middle lobe, the other in the lower lobe). Three weeks before the surgery, the alpha-adrenergic receptor blocker, phenoxybenzamine (10 mg bid), was used for normalizing blood pressure and heart rate. The patient underwent left adrenalectomy to remove the tumor. The histopathological examination showed that the tumor was pheochromocytoma, and the Pheochromocytoma of the Adrenal Gland Scaled Score (9) is 14. After the surgery, hypertension and palpitations were relieved. CT scans were performed annually, and the lung metastases grew gradually without any symptoms. Three years later (October 2013), contrast-enhanced CT showed increased and enlarged nodules in both lungs. There were five enhanced nodules, including a 2.5 × 2.3-cm lesion in the right lower lobe, a 2.2 × 1.8-cm lesion in the right middle lobe, and three 1.0 × 1.0-cm lesions in the left lobes (**Figure 1A**). Hypertension reappeared with the blood pressure ranged from 160/100 mmHg to 180/110 mmHg, but palpitations were not found. Biochemical testing showed elevated levels of plasma-free normetanephrine (4937 pg/mL, normal range <160 pg/mL) and 24-hour urine metanephrine (633 μg, normal range <580 μg). Additionally, CT-guided percutaneous biopsy of the pulmonary nodule was performed. Histopathological examination confirmed the diagnosis of metastatic pheochromocytoma (**Figure 2**). For multilocal lesions in bilateral lungs, surgical resection was not suitable, and local minimally invasive therapy might take more benefits than systemic therapy. Considering the fewer side effects and cost, the patient chose the ^125^I seed implantation.

The patient received phenoxybenzamine (10 mg bid) to control blood pressure 2 weeks before the procedure and 2 months after the procedure. Percutaneous ^125^I seed implantation was performed under the CT guidance, and local anesthesia was used during the procedure. The implantation plan was determined according to the treatment planning system based on the patient’s enhanced CT data. The activity of each implanted ^125^I seed was 0.7 mCi, and the prescription dose of each lesion was 120-140 Gy (10). A total of 80 seeds (32 in the lesion of the right lower lobe, 18 in the lesion of the right middle lobe, 10 in each lesion of the left lobe) were implanted into the target lesions through 18 G puncture needles. No pulmonary hemorrhage, pneumothorax, or chest pain was found during the perioperative period. The patient’s secondary hypertension was relieved with the blood pressure of ≤ 140/90 mmHg 2 months later. Levels of 24-hour urine metanephrine and plasma-free normetanephrine returned to normal 2 months and 6 months later, respectively (**Table 1**). CT scans were performed per 6 months for the first year. The lesions achieved partial response according to Response Evaluation Criteria in Solid Tumors version 1.1 (RECIST v1.1) 6 months later, and further shrank 1 year later (**Figure 1B**). Thereafter, the patient was followed up with CT scans annually and her disease remained stable for 7 years as of the last follow-up on October 25, 2020.

**Table 1:** Biochemical laboratory findings before and after ^125^I seed implantation.

Indicators	Pre	2 months	6 months	1 year	Normal range
Plasma-free normetanephrine, pg/mL	4937	1081	146	52	<160
Urine metanephrine, μg/24h	633	316	121	90	<580

## Discussion

Pheochromocytoma is a rare tumor in clinics, the incidence of pheochromocytoma is about 2 to 5 cases per million person-years (11). Nearly one-fifth of patients with pheochromocytoma experience disease progression at a slow rate. Compared with most malignant tumors, pheochromocytoma has a relatively better prognosis, but metastatic pheochromocytoma usually has a poor prognosis. A systematic review (12) showed that overall and 5-year mortality rates for metastatic pheochromocytoma were 53% and 42%, respectively. Pheochromocytoma can release catecholamines and their metabolites, which lead to adrenergic symptoms, such as hypertension, headache, and palpitations. If the tumor is not removed, life-threatening hypertension and cardiac disorders may happen (1). In addition, metastases could also result in lesion-related mass effect, commonly leading to pain and compression of surrounding structures. For the symptomatic or rapidly progressive metastatic pheochromocytoma, treatment is necessary, and several local or systemic therapies have been successfully used (5).

Surgical resection is the preferred treatment for patients with resectable metastatic pheochromocytomas, and a minimally invasive approach is recommended (13). However, in some cases, such as those with multiple local lesions, patients are not suitable for surgery. Percutaneous interventional techniques, including radiofrequency ablation (RFA), cryoablation, percutaneous ethanol injection (PEI), and cementoplasty, were also reported to be efficient for local control and symptom relief in metastatic pheochromocytomas. A recent study (14) reported the efficacy of ablative therapy for metastatic pheochromocytoma and paraganglioma (PPGL). Thirty-one patients with 123 lesions underwent a total of 69 ablation sessions (42 RFA, 23 cryoablation, and 4 PEI). Radiographic local control was achieved in 86% of the ablated lesions (86% with RFA, 86% with cryoablation, and 50% with PEI), and improvement in adrenergic symptoms or metastasis-related pain was achieved in 92% of procedures. Liu et al. (15) reported a case on malignant pheochromocytoma with sacrum metastases, which were treated with cementoplasty after embolization of the internal iliac artery. Although cement-related spinal canal stenosis occurred, the patient’s neurological deficits improved after circumferential spinal cord decompression.

As another percutaneous interventional technique, ^125^I seed implantation was first used for the treatment of prostate cancer in the 1970s, and it has been the first-line treatment for localized prostate cancer (16). In recent years, image-guided ^125^I seed implantation has also been used for the treatment of various primary or metastatic solid tumors, such as lung cancer, liver cancer, bone cancer, and lymph node metastases (10, 17–19). Since the common metastatic sites of pheochromocytoma are also the common implanted sites of ^125^I seeds, we assumed that ^125^I seed implantation could become a feasible treatment in metastatic pheochromocytoma. Our case illustrated that percutaneous ^125^I seed implantation can be safely used to treat metastatic pheochromocytoma in the lung. Thermal ablation techniques such as RFA have the defect of invisible spherical ablation margin during the procedure, due to which adjacent crucial organs may be damaged or tumor may be undertreated (20). The implanted ^125^I seeds are arranged according to the shape of the tumor. Thus, we believe ^125^I seed implantation could be an alternative treatment for thermal ablation and is more suitable for patients with lesions adjacent to crucial organs.

Pheochromocytoma is sensitive to radiation therapy. The targeted radiolabeled carrier, high-specific-activity (HAS) ^131^I-metaiodobenzylguanidine (^131^I-MIBG) or ^177^Lutetium-DOTA-Tyr3-Octreotate (^177^Lu-DOTATATE), was recommended for the treatment of unresectable metastatic pheochromocytoma if tumors are positive on MIBG scan or somatostatin receptor imaging. As reported in a phase 2 multicenter trial (21) investigating HAS ^131^I-MIBG to treat patients with metastatic or unresectable PPGL, partial response and stable disease were achieved in 23% and 68% of patients respectively, and the median overall survival was as long as 36.7 months. However, hematologic toxicity (90%) and gastrointestinal adverse events, such as nausea (78%) and vomiting (53%), were common. A study of 30 patients with metastatic or unresectable PPGL treated with ^177^Lu-DOTATATE displayed similar results (22). Partial response and stable disease were achieved in 23% and 67% of patients respectively, and grade 3 or 4 hematologic toxicity was observed in 20% of patients. EBRT was another recommended palliative therapy for metastatic pheochromocytoma. As literature (23, 24) showed, the radiographic local control rate was 81%-87% and the symptom improvement rate was 81%-94% in patients with metastatic PPGL treated with EBRT.

^125^I seed implantation is a modality of internal radiation therapy, which releases continuous low-dose-rate x-ray and γ-ray. Although the type of radioactive rays and the form of irradiation are different from the radiopharmaceutical agents and EBRT, the pulmonary metastatic pheochromocytoma in our case showed good sensitivity to ^125^I seed radiation. Compared with targeted radiopharmaceutical agents and EBRT, ^125^I seed implantation provides the advantage of delivering higher doses of radiation to the tumor with relative sparing of surrounding normal tissues. The cumulative dose achieved 120-140 Gy in each pulmonary lesion in this case, while the median cumulative dose was only 24 Gy in EBRT (23). The high cumulative doses delivered by ^125^I seeds could be the main reason for such good efficacy: all 5 pulmonary lesions shrank significantly, the patient’s hypertension was relieved, and no new metastasis was found. Moreover, in this case, no radiation-related adverse event was found during follow-up. Damage or stress to the tumor could cause the release of large amounts of catecholamines, aggravating adrenergic symptoms, even resulting in life-threatening hypertension (13). The half-life period of ^125^I seed is 60.2 days, approximately half of the dose is delivered within 2 months. In our opinion, to reduce catecholamine-related risks, it is reasonable to preventively use the alpha-adrenergic receptor blocker for at least 2 months after implantation.

In conclusion, our case illustrated that ^125^I seed implantation had a powerful anti-tumor effect on metastatic pheochromocytoma with pulmonary metastases. We propose that ^125^I seed implantation should be further evaluated as an alternative local therapy in patients with unresectable metastatic pheochromocytoma.

## Data Availability Statement

The original contributions presented in the study are included in the article/supplementary material. Further inquiries can be directed to the corresponding author.

## Ethics Statement

Written informed consent was obtained from the individual(s) for the publication of any potentially identifiable images or data included in this article.

## Author Contributions

WH conceived the idea of this case report and was responsible for the treatment. HS, WQ, and YY participated in the treatment. CW, BS, and HW participated in the follow-up and data collection. HS and CW wrote the first draft of the manuscript. All authors contributed to the article and approved the submitted version.

## Conflict of Interest

The authors declare that the research was conducted in the absence of any commercial or financial relationships that could be construed as a potential conflict of interest.

## Publisher’s Note

All claims expressed in this article are solely those of the authors and do not necessarily represent those of their affiliated organizations, or those of the publisher, the editors and the reviewers. Any product that may be evaluated in this article, or claim that may be made by its manufacturer, is not guaranteed or endorsed by the publisher.
